# Supplementary material for: Nutritional quality of retail food purchases is not associated with participation in the Supplemental Nutrition Assistance Program for nutrition-oriented households
Source: PLoS One. 2020 Dec 18;15(12):e0240263. doi: 10.1371/journal.pone.0240263 (PMC7748149; doi:10.1371/journal.pone.0240263)
Supplement: S1 File — (DOCX) [file pone.0240263.s001.docx]

**Appendix A. Food price index construction using the weighted product dummy method**

Accurate measurement of food prices is key to interpreting the household’s purchasing behavior as market prices fluctuate over time and across regions, as well as in characterizing the constraints that a household faces ^1^. In this paper, we construct the household-level price indexes for starred and unstarred foods, using a weighted product dummy method. This approach is a modified version of the weighted country product dummy method used for international price comparisons ^2,3^. This method projects product prices onto a set of location, time, household, and Universal Product Code (UPC) dummies by running the following weighted regression:

$lnP_{ich}=\alpha PSU_{h}+\beta WEEK_{h}+\gamma HH_{h}+\theta{UPC}_{i}+\varepsilon_{ich}\begin{matrix} & c=1, 2 \end{matrix}$.

The subscript *i* indexes the UPC, *c* indicates whether *i* is starred (*c* = 1) or unstarred (*c* = 2), and *h* denotes the household. $P_{ich}$ is the price of UPC $i$ in star category $c$ purchased by household $h$. *PSU* is primary sampling unit (PSU) dummy, *WEEK* is weekly dummy, *HH* is the household dummy, $UPC$ is the UPC dummy, and $\varepsilon_{ich}$ is the error term*.* The weight in the regression is the budget share of each UPC in star category *c* purchased by household *h*. The argument for weighting is that products with larger budget shares should count more in the price index calculations. Therefore the fitted price index model should weigh prices of more popular products higher than products purchased less frequently.

We use the estimated coefficients of PSU, week, and household dummies to construct the household-level price indexes for starred foods and unstarred foods. Product fixed effects are differenced out by omitting coefficient estimates on the UPC dummies from the price index construction. This method removes the portion of variation in household-reported prices that are caused by product heterogeneity (i.e., product type, variety and quality) within a star category, which results in price indexes that better measure market price variation across locations and households.

**Appendix B Supplemental Tables**

S1 Table. Associations between nutritional quality and covariates among low-income households

|  | **Guiding Stars rating** | | | |
| --- | --- | --- | --- | --- |
|  | **Nutrition-oriented households** | | **Less nutrition-oriented**  **households** | |
|  | Coefficient | Std. Err. | Coefficient | Std. Err. |
| SNAP participation (Yes=1) | 0.016 | 0.053 | -0.104^**^ | 0.039 |
| WIC participation (Yes=1) | 0.003 | 0.066 | 0.015 | 0.034 |
| Food price ratio | -0.107 | 0.080 | -0.084^**^ | 0.039 |
| Food insecure (Yes=1) | -0.093 | 0.073 | 0.043 | 0.044 |
| Standardized food expenditure | 0.038 | 0.031 | 0.014 | 0.012 |
| Rural (Yes=1) | -0.026 | 0.070 | -0.056 | 0.039 |
| Household size | -0.046^*^ | 0.026 | -0.016 | 0.012 |
| Share with children | -0.381^***^ | 0.122 | -0.283^***^ | 0.070 |
| Share with older adults | -0.060 | 0.215 | 0.106^*^ | 0.059 |
| Share with ≥1 Hispanic member | 0.125^*^ | 0.073 | 0.125^***^ | 0.040 |
| Share with ≥1 obese member | -0.086 | 0.112 | -0.039 | 0.043 |
| Share with ≥1 smoker | -0.396^***^ | 0.104 | -0.192^***^ | 0.049 |
| Share with ≥1 member in poor health | -0.161 | 0.209 | 0.027 | 0.082 |
| Household financial condition | -0.0004 | 0.097 | 0.015 | 0.037 |
| Own house (Yes=1) | -0.066 | 0.069 | 0.059 | 0.048 |
| Food pantry/food bank (Yes=1) | -0.097 | 0.115 | -0.086^**^ | 0.040 |
| PR’s highest education | 0.019 | 0.034 | 0.017 | 0.013 |
| Constant | 1.048^***^ | 0.214 | 0.775^***^ | 0.072 |
| *N* | 471 |  | 1,747 |  |
| *R^2^* | 0.184 |  | 0.129 |  |

*^a^* Boldface indicates statistical significance (*** p<0.01, ** p<0.05, * p<0.1).

*^b^* The estimates use sample weights and control for survey design. Nutrition-oriented households are those who reported searching for nutrition information online in the last two months. Less nutrition-oriented households are those who did not.

S2 Table. Associations between HEI-2010 score and covariates among low-income households

|  | **HEI-2010 score** | |
| --- | --- | --- |
|  | Coefficient | SE |
| SNAP participation (Yes=1) | -2.106^*^ | 1.063 |
| SNAP×NutritionSearch | 1.365 | 2.295 |
| NutritionSearch (Yes=1) | 1.476 | 1.679 |
| WIC participation (Yes=1) | 1.364 | 0.851 |
| Food price ratio | -3.251^***^ | 0.798 |
| Food insecure (Yes=1) | -1.061 | 1.022 |
| Standardized food expenditure | 2.563^***^ | 0.623 |
| Rural (Yes=1) | -0.952 | 1.353 |
| Household size | -1.215^***^ | 0.317 |
| Share with children | -4.712^***^ | 1.487 |
| Share with older adults | -0.630 | 1.539 |
| Share with ≥1 Hispanic member | 2.562^**^ | 1.182 |
| Share with ≥1 obese member | -1.524 | 1.827 |
| Share with ≥1 smoker | -9.015^***^ | 1.133 |
| Share with ≥1 member in poor health | 3.320 | 2.437 |
| Household financial condition | 1.676 | 1.295 |
| Own house (Yes=1) | 2.265^*^ | 1.222 |
| Food pantry/food bank (Yes=1) | 0.546 | 1.879 |
| PR’s highest education | 0.602^*^ | 0.315 |
| Constant | 55.173^***^ | 1.926 |
| *N* | 2,218 |  |
| *R^2^* | 0.141 |  |

*^a^* Boldface indicates statistical significance (*** p<0.01, ** p<0.05, * p<0.1).

*^b^* All estimates use sample weights and control for survey design.

S3 Table. Associations between nutritional quality and covariates among low-income households

|  | **Guiding Stars rating** | |
| --- | --- | --- |
|  | Coefficient | Std. Err. |
| SNAP participation (Yes=1) | -0.120** | 0.052 |
| SNAP×NutritionLabel | 0.069 | 0.056 |
| NutritionLabel (Yes=1) | 0.008 | 0.042 |
| WIC participation (Yes=1) | 0.010 | 0.030 |
| Food price ratio | -0.088** | 0.037 |
| Food insecure (Yes=1) | 0.014 | 0.041 |
| Standardized food expenditure | 0.018 | 0.013 |
| Rural (Yes=1) | -0.050 | 0.042 |
| Household size | -0.024** | 0.011 |
| Share with children | -0.294*** | 0.061 |
| Share with older adults | 0.082 | 0.051 |
| Share with ≥1 Hispanic member | 0.128*** | 0.040 |
| Share with ≥1 obese member | -0.051 | 0.040 |
| Share with ≥1 smoker | -0.222*** | 0.050 |
| Share with ≥1 member in poor health | -0.015 | 0.075 |
| Household financial condition | 0.008 | 0.033 |
| Own house (Yes=1) | 0.036 | 0.043 |
| Food pantry/food bank (Yes=1) | -0.092** | 0.042 |
| PR’s highest education | 0.017 | 0.014 |
| Constant | 0.830*** | 0.066 |
| *N* | 2,218 |  |
| *R^2^* | 0.130 |  |

*^a^* Boldface indicates statistical significance (*** p<0.01, ** p<0.05, * p<0.1).

*^b^* The estimates use sample weights and control for survey design.

S4 Table. Number of nutrition-oriented and less nutrition-oriented by SNAP status.

|  | **SNAP households (N=1,184)** | **Non-SNAP households (N=1,034)** |
| --- | --- | --- |
| **Whether had online nutrition search** |  |  |
| Nutrition-oriented | N=247 | N=224 |
| Less nutrition-oriented | N=937 | N=810 |
| **Whether used nutrition facts label** |  |  |
| Nutrition-oriented | N=672 | N=660 |
| Less nutrition-oriented | N=512 | N=374 |

**REFERENCES**

1. McKelvey C. Price, unit value, and quality demanded. *Journal of Development Economics.* 2011;95(2):157-169.

2. Deaton A, Dupriez O. Purchasing power parity exchange rates for the global poor. *American Economic Journal: Applied Economics.* 2011;3(2):137-166.

3. Rao DSP. On the equivalence of Weighted Country‐Product‐Dummy (CPD) method and the Rao‐system for multilateral price comparisons. *Review of Income and Wealth.* 2005;51(4):571-580.
