# Supplementary material for: Nutritional quality of retail food purchases is not associated with participation in the Supplemental Nutrition Assistance Program for nutrition-oriented households
Source: PLoS One. 2020 Dec 18;15(12):e0240263. doi: 10.1371/journal.pone.0240263 (PMC7748149; doi:10.1371/journal.pone.0240263)
Supplement: Attachment — Submitted filename: Response to Reviewers.docx [file pone.0240263.s002.docx]

**Response to Reviewer 1’ comments**

Before addressing your comments, we would like to thank you for spending time on the review and for the helpful suggestions. We have made every effort to address your concerns. Our responses are outlined below your comments in a point-by-point manner. For ease of review, your comments are reproduced in *italic*; our responses are provided in the normal font. We have also incorporated your suggestions and our responses into the revised manuscript; changes are marked in blue color. We hope that our revisions have addressed all your concerns. But we stand ready to make further modifications should you have any further concerns or suggestions.

*Reviewer #1: By mainly use of multiple regression analysis s seen on lines 110 to 138, the investigators concluded that SNAP participation was associated with lower nutritional quality of food purchases among less nutrition-oriented households, but not among nutrition-oriented households. The lack of such an association for nutrition-oriented participants suggests that the intended nutrition benefit of placing restrictions on purchasing unhealthy foods with SNAP benefits may not reach this subgroup of SNAP population. The statistical applications are simple. However, the presentation of the results is very confusing. Specifically:*

*1. The results are presented by low income household and the text also discusses results by nutritional attitude and then performs a robustness analysis and demonstrates the consistency of the results. The tables in the text are mostly results by low income households and Table 2 appears to attempt to show the results by less nutritional vs. nutritional oriented household by manipulation of the regression coefficients. Is one to assume that all the households in the study are low income?*

**Response**: Thanks for your comments. Our analysis focused exclusively on low-income households whose income below 185% of the Federal Poverty Line (FPL). Our sample includes 2,218 low-income households, 1,184 households are SNAP participants and 1,034 households are nonparticipants. A description of the study sample was in line 93-97. In the current version, we rephrased the sentences in line 93-94 to further clarify. Table 3 (formerly table 2) reports our main regression results and has the phrase “among low-income households” in the table title. We are open to additional suggestions on how we may improve clarity of the sample selection.

*2. Except for presentation of the covariates in the multiple regression format there are no tables in the paper showing a side by side comparison of the less nutrition vs. nutrition oriented comparisons which would be of interest. Also, Table 2 with the manipulation of the -0.097+0.089 on line 176 can be confusing to a non statistically oriented reader. Also what is SNAP*Nutrition Search? Is that an interaction term and how does it differ from Nutrition Search (Yes=1)?*

**Response**: Thanks for your valuable suggestions and comments. We have taken the following two steps to improve the presentation.

First, we add a new table 2 (page 13), shown below, that compares nutrition quality of retail food purchases between SNAP and non-SNAP households by nutritional attitude. Compared to nonparticipants, SNAP participants on average had a 0.2- point lower Guiding Stars rating than low-income nonparticipants among less nutrition-oriented households, while the difference is smaller and statistically insignificant among nutrition-oriented households. Similarly, for less nutrition-oriented households, SNAP households had a 4.274- point lower HEI-2010 score than nonparticipants. In comparison, for nutrition-oriented households, the difference between SNAP and non-SNAP households was smaller and not significant.

**Table 2 Comparison of Guiding Stars rating and HEI-2010 score between SNAP and low-income non-SNAP households by nutrition attitude.**

|  | **SNAP**  **participants**  **(1)** | **Income - eligible**  **Non-SNAP participants**  **(2)** | **Mean**  **Difference**  **(1) - (2)** |
| --- | --- | --- | --- |
| **Guiding Stars rating** |  |  |  |
| Nutrition-oriented | 0.631 (0.038) | 0.698 (0.058) | -0.067 |
| Less nutrition-oriented | 0.538 (0.025) | 0.739 (0.025) | -0.200^***^ |
| **HEI-2010 score** |  |  |  |
| Nutrition-oriented | 50.413 (1.339) | 52.203 (1.636) | -1.790 |
| Less nutrition-oriented | 45.976 (0.574) | 50.250 (0.845) | -4.274^***^ |

*^a^* Calculation of weighted means (standard errors in parentheses) accounts for the survey design of FoodAPS.

*^b^* The *t*-test is used to test the difference between SNAP households and nonparticipants. Boldface indicates statistical significance (*** p<0.01, ** p<0.05, * p<0.1).

Second, table 3 (formerly table 2) shows the association of SNAP with the Guiding Stars rating of food-at-home purchases. In the previous version, “SNAP*NutritionSearch” was the interaction between SNAP participation and NutritionSearch. In the current version, we switched to “SNAP×NutritionSearch” and added a footnote to table 3 to be more clear that this is an interaction term. This interaction term captured the heterogeneity in the SNAP–nutritional quality association that can be tracked to a household’s nutrition attitude (discussed in lines 223-225). The coefficient (-0.009) on the NutritionSearch variable measures the difference in the level of the dependent variable between households who searched for nutrition information and those who did not, when SNAP=0. The coefficient (0.089) on the interaction term measures the difference in the slope of the SNAP participation–nutritional quality association. In other words, 0.089 is the extent to which NutritionSearch moderates the SNAP-nutritional quality relationship, which is the main relationship of interest for this study. In general, when using an interaction term as a covariate in a regression, it is important to include the elements of the interaction variable as stand-alone covariates in addition to the interaction term to avoid spurious results.

To interpret the results, for less nutrition-oriented households (whose NutritionSearch=0), SNAP participation is associated with a 0.097-point (*p*=0.018) lower Guiding Stars rating. For nutrition-oriented households (whose NutritionSearch=1), SNAP participation is associated with a 0.008-point (*p*=0.890) lower Guiding Stars rating. The −0.008 is the sum of −0.097 and 0.089. We rewrote line 228-230 to better describe the calculation.

*3. How much of the variation in the dependent measures are being explained by the regressions? There are no R-squares in the results?*

**Response**: We added the R-squared values in the results of all regressions. Specifically,

When we regress household’s Guiding stars rating on SNAP and other covariates (Table 3, on page 15): R^2^ =0.130

When we regress household’s HEI-2010 score on SNAP and other covariates (Supplemental file, Table S2): R^2^=0.141

We also report R^2^ of other regressions, please refer to：

Table 5 on page 18-19 in the revised manuscript.

Table S1, Table S3 in the supplemental file.

**Response to Reviewer 2’ comments**

Before addressing your comments, we would like to thank you for spending time reading the manuscript and providing the helpful suggestions. We have made every effort to address your concerns. Our responses are outlined below your comments in a point-by-point manner. For ease of review, your comments are quoted in *italic*; our responses are provided in the normal font. We have also incorporated your suggestions and our responses into the revised manuscript; changes we made are marked in blue. We hope that our revisions have addressed all your concerns. But we stand ready to make further modifications should you have any further concerns or suggestions.

*Reviewer #2: This article addresses an important issue (is the intended restrictions on purchasing unhealthy foods with SNAP of nutrition benefit?), however this paper only partially answers this. While the paper is comprehensive, I found at times I had to carefully reread sections to understand the paper fully. There are some points that need to be clarified (stated below).

Abstract:
FoodAPS - State this in full.*

**Response**: Thanks! We revised this sentence (line 9-10).

*16: among*
**Response**: Thanks! We corrected this word (line 18).

*Income-eligible nonparticipants. As someone not familiar with the details of SNAP, it would be useful to explain the income criteria for SNAP and describe the income-eligible non-participants. Why don't they receive SNAP? Are they different to the SNAP participants (partly addressed in results)?*

**Response**: Thanks for your suggestions. We added the information about the income criteria for SNAP and describe some characteristics of income-eligible non-participants in the revised manuscript. Please see lines 34-43.

(1) *income criteria for SNAP*:

There are two basic pathways for financial eligibility for the SNAP program: 1). Meeting federal eligibility requirements: households must have net income at or below 100% of the Federal poverty thresholds (FPL). Besides, households without an elderly or disabled member must have monthly gross income at or below 130% FPL. 2). Or being automatically eligible for SNAP based on being eligible for or receiving benefits from other safety net programs (e.g., the Temporary Assistance for Needy Families program): this requirement varies by states. The gross income limit for households without an elderly or disabled member varies between 130% and 200% FPL. For more detailed information, please refer to Aussenberg and Falk (2019).

FoodAPS data is nationally representative of SNAP households and nonparticipant households in three income groups: those with incomes below 100% FPL, 100 % -185% FPL, and ≥185% FPL. Following the literature (e.g., Taylor and Villas-Boas, 2016), we treated non-SNAP participating households with incomes below 185% FPL as “eligible non-SNAP households”.

References:

Aussenberg, Randy Alison, and Gene Falk. "The Supplemental Nutrition Assistance Program (SNAP): Categorical Eligibility." Congressional Research Service Reports (2019).

Taylor, Rebecca, and Sofia B. Villas-Boas. "Food store choices of poor households: A discrete choice analysis of the National Household Food Acquisition and Purchase Survey (FoodAPS)." *American Journal of Agricultural Economics* 98.2 (2016): 513-532.

(2) *describe the income-eligible non-participants. Why don't they receive SNAP? Are they different to the SNAP participants (partly addressed in results)?*

Previous studies showed that eligible working-poor families and elderly had low SNAP participation rates (Greenstein et al., 2018). Some potential reasons are: 1. the enrollment and renewal procedures of SNAP are complicated and time-consuming (e.g., require multiple trips to the welfare office). 2. Some people didn’t realize that they were eligible for SNAP or were opposed to taking public assistance. Some states have worked on simplifying SNAP policies and improved their outreach to eligible households to increase SNAP enrollment.

One review (Pinard et al., 2017) summarized the characteristics of eligible non-SNAP households, for example: compared to SNAP households, nonparticipants have higher incomes and assets, have fewer children, less likely to have disabled members, and more likely to have older adults. Also, single-parent households are more likely to receive SNAP than married or partnered households

We observed similar results, as shown in table 1, we found that income-eligible non-**SNAP households had a smaller proportion of children and a larger proportion of older adults. Also, relative to SNAP households, nonparticipants were more likely to own a house and were more satisfied with their financial condition.**

References:

Robert Greenstein, Brynne Keith-Jennings, and Dorothy Rosenbaum. " Factors Affecting SNAP Caseloads." Washington, DC: Center on Budget and Policy Priorities (2018).

Pinard, C. A., et al. "What factors influence SNAP participation? Literature reflecting enrollment in food assistance programs from a social and behavioral science perspective." Journal of Hunger & Environmental Nutrition 12.2 (2017): 151-168.

*In-text citations: Be consistent with space before the citation*

**Response**: Thanks! We checked the manuscript to make sure space before citations were consistent.

*Introduction:
55: It is not clear to me how this study controls for the influences of food prices? This may be stated in the paper but there is likely to be other readers who require this to be clearer.*

**Response**: Thanks for your comments. We controlled for food prices by including household-specific price ratio into the model, which was calculated as the overall price of healthy (Guiding stars: 1 to 3 ratings) foods divided by the overall price of less healthy (0 ratings) foods. For more detailed information about the construction of the household’s price ratio, please see appendix A in the supplemental file.

We add this information to the “Introduction” section, please see lines 69-72.

*60: research studies*
**Response**: Thanks! We revised this sentence, please see line 74-75.

*Methods:
68: Is the age of the dataset a limitation? Is there no recent data?*

**Response**: Thanks for your comments. FoodAPS was fielded between April 2012 and January 2013. There is no recent data released. The next survey, FoodAPS-2, will be fielded in 2022. This counts one limitation, and we added to the “Limitations” section. Please see lines 372-376.

More information about FoodAPS, please refer to:

<https://www.ers.usda.gov/data-products/foodaps-national-household-food-acquisition-and-purchase-survey/background/>

*74: "Oils ....*

**Response**: Thanks! We revised this, please see line 89.

*90: Please explain more about the Guiding Stars. Is this a common nutrient profiling tool in the US? What increments are used? Is there a commonly accepted cut-off for unhealthy and less healthy?*

**Response**: Thanks for your comments. We added more information on the Guiding Stars program and please see lines 106-108, 116-118.

(1) Guiding stars is a nutrition guidance program that was first implemented at the Hannaford supermarket chain in September 2006. The purpose of this program is to help consumers quickly identify foods and beverages that are consistent with dietary recommendations. Guiding Stars is now available at major grocery chains and food service facilities at thousands of locations across the U.S., and it is growing as a leading independent nutrition guidance program.

(2) The program evaluates the nutrient content of foods and beverages using nutrients displayed on the Nutrition Facts label and the list of ingredients printed on product packaging. For foods that are not labeled, such as fruits and vegetables, the program uses information obtained from other nutrition databases. The program is driven by an algorithm that generates weighted scores based on points debited for trans fat, saturated fat, cholesterol, sodium, and added sugars and credited for vitamins and minerals, fiber, and whole grains.

(3) Guiding Stars does not rank foods as “unhealthy” or “less healthy” and there is not a uniform cut-off for a product to be healthy or unhealthy. Instead, food items are ranked as “unstarred”, one star, two stars, and three stars. “Unstarred” means the food item does not meet the nutritional criteria to receive a Guiding Star. One star means good nutrition value, two stars means better nutritional value, and three stars means the best nutritional value.

To assign the Guiding Stars rating, grocery items first receive scores based on the amounts of various nutrients in the product. Then, foods with negative scores were treated with unstarred food. Foods with positive scores can be further assigned to different stars rating.

*118: You use households that had online nutrition search as a key measure. Has this been used elsewhere? It is good that an alternative measure is also used but has either been verified against a comprehensive tool to assess nutrition attitude?*

**Response**: Thanks for your comments.

(1) Previous studies found that a nutrition attitude is associated with diet quality (e.g., Aggarwal et al., 2013). Based on the variables included in FoodAPS, we used online nutrition information search as a key measure of nutrition attitude. One study (Lê et al., 2013) used a similar measurement to assess the nutrition attitude. They investigated attitudes toward healthy eating and used “Searching for information about food” as an important indicator to assess nutrition attitude. Searching nutrition information through the internet is an important way to gain nutrition knowledge which reflects the consumer’s nutrition attitude.

(2) Compared to the two measures used in our study, there isn’t a more comprehensive variable to assess nutrition attitude in the FoodAPS dataset.

To look at the robustness of our results, we used nutrition fact labels utilization as another measurement to assess the nutrition attitude. In previous studies, nutrition label use has been linked to consumer’s nutrition attitude (e.g., Cowburn and Stockley, 2005), and many studies investigate the relationship between nutrition label use and diet quality (e.g., Buyuktuncer, et al. 2018; Kollannoor-Samuel, et al. 2016).

The results are robust using two measures of nutrition attitude: SNAP was associated with lower nutritional quality of food purchases among less nutrition-oriented households, while this association was not significant among nutrition-oriented households.

References

Aggarwal A, Monsivais P, Cook AJ, Drewnowski A. Positive attitude toward healthy eating predicts higher diet quality at all cost levels of supermarkets. J Acad Nutr Diet. 2014;114(2):266-272. doi:10.1016/j.jand.2013.06.006

Lê, J., et al. "Attitudes toward healthy eating: a mediator of the educational level–diet relationship." European Journal of Clinical Nutrition 67.8 (2013): 808-814.

Cowburn, G., & Stockley, L. (2005). Consumer understanding and use of nutrition labelling: A systematic review. Public Health Nutrition, 8(1), 21-28. doi:10.1079/PHN2004666

Buyuktuncer, Z.; Ayaz, A.; Dedebayraktar, D.; Inan-Eroglu, E.; Ellahi, B.; Besler, H.T. Promoting a Healthy Diet in Young Adults: The Role of Nutrition Labelling. *Nutrients* 2018, *10*, 1335.

Kollannoor-Samuel, et al., Nutrition facts panel use is associated with higher diet quality and lower glycated hemoglobin concentrations in US adults with undiagnosed prediabetes, *The American Journal of Clinical Nutrition*, Volume 104, Issue 6, December 2016, Pages 1639–1646, <https://doi.org/10.3945/ajcn.116.136713>

*Results:
141: Add that the differences were statistically significant*

**Response**: Thanks for the suggestion! We add a significant level (*p*<0.001) to the sentence, please see line 166, and line 171.

*156: As SNAP households seem to have different characteristics to non-SNAP, is it these differences that explain the differences observed in the study? You may have stated this in the results but it needs to be clearly addressed.*

**Response**: Thanks for this suggestion. SNAP households have different characteristics from nonparticipants. Previous studies (e.g. Pinard, et al. 2017) showed that compared to non-SNAP households, SNAP households have lower incomes and assets, have more children, more likely to have disabled members, and less likely to have older adults. Also, single-parent households are more likely to receive SNAP than married or partnered households.

Our study observed similar results, as shown in table 1, we found that **SNAP households had a smaller proportion of children and a larger proportion of older adults. Also, relative to SNAP households, nonparticipants were more likely to own a house and were more satisfied with their financial condition.**

**Given the different characteristics between the two group populations, we controlled for these observed characteristics in our regression analysis. After controlling these characteristics, we found that** SNAP was associated with lower nutritional quality of food purchases among less nutrition-oriented households, while this association was not significant among nutrition-oriented households.

We added more information in the revised manuscript, please see lines 172-175, and lines 221-222).

References:

Pinard, C. A., et al. "What factors influence SNAP participation? Literature reflecting enrollment in food assistance programs from a social and behavioral science perspective." Journal of Hunger & Environmental Nutrition 12.2 (2017): 151-168.

*Nutrient density has been used as a measure. Can you also assess overall energy intake? This is obviously key to obesity.*

**Response**: Thanks for this suggestion. In the revised manuscript:

1. We compared per capita energy acquired per 7 days between SNAP households and nonparticipants, and added this information in Table 4 (please see page 16). Compared to nonparticipants, SNAP households on average had more energy acquired per person (2710 kcal) over the one-week survey period (line 247-248).

2. We looked at the association between covariates and per capita energy acquired among low-income households and added this information in Table 5 (page 18) and corresponding interpretation (line 272-275). SNAP participation was associated with more per capita energy consumption among less nutrition-oriented households, while this association was not statistically significant among nutrition-oriented households at conventional levels of significance (*p*=0.126).

Our result is consistent with previous studies, for example, Leung and Villamor (2011) suggested that SNAP participation was associated with more energy intake, which leads to a higher risk of obesity.

Reference:

Leung, Cindy W., and Eduardo Villamor. "Is participation in food and income assistance programs associated with obesity in California adults? Results from a state-wide survey." Public health nutrition 14.4 (2011): 645-652.

*The results do not present actual numbers of participants anywhere. It would be useful to know how many households were considered 'nutrition-oriented' for SNAP and non-SNAP participants by both methods.*

**Response**: Thanks for this suggestion. We added the number of the observations in the revised manuscript: page 13 (line 201-203), Tables 1, 3, 5.

The following table shows the number of ‘nutrition-oriented' and ‘less nutrition-oriented’ for SNAP and non-SNAP participants using both measures of nutrition attitude. We added this table in the supplemental file (Table S4) and mentioned this in the manuscript (lines 203-204, and lines 305-306).

S4 Table. Number of nutrition-oriented and less nutrition-oriented by SNAP status.

|  | **SNAP households (N=1,184)** | **Non-SNAP households (N=1,034)** |
| --- | --- | --- |
| **Whether had online nutrition search** |  |  |
| Nutrition-oriented | N=247 | N=224 |
| Less nutrition-oriented | N=937 | N=810 |
| **Whether used nutrition facts label** |  |  |
| Nutrition-oriented | N=672 | N=660 |
| Less nutrition-oriented | N=512 | N=374 |

*The robustness check is useful. How many participants were considered 'nutrition-oriented' by both methods? How many participants had low HEI scores and low star scores?*

**Response**: Thanks for these questions!

**1. “***How many participants were considered 'nutrition-oriented' by both methods?”*

**(1) When we used households’ responses** on whether they searched online nutrition information in the last 2 months to **measure their nutrition** attitude: 471 households were nutrition-oriented households and 1,747 households were less nutrition-oriented households. We added this information on page 13 (line 201-203).

(2) When we used households’ responses on whether they used the nutrition facts label to measure their nutrition attitude: 1,332 households were nutrition-oriented households and 886 households were less nutrition-oriented households (line 303-305).

2. *“How many participants had low HEI scores and low star scores?”*

We calculated the HEI-2010 score and Guiding Stars rating at the household level, and both of them are continuous variables. As shown in Table 1, overall, the mean HEI-2010 score is 49.17 (SE=0.505), and the mean **Guiding Stars rating is** 0.664 (SE=0.016)**.**

We didn’t have a specific cut-off value to define low HEI scores or low Guiding Stars rating of a household. The table below shows the distributions of HEI scores and Guiding Stars rating among all low-income households. We can see that compared to the mean HEI-2010 score of 49.174, over 50% of households had a lower HEI-2010 score, similarly, over 50% of households had a lower star rating than the average star rating of 0.664.

Also, we can see that 25% of households had HEI-2010 score lower than 38.4 points and had Guiding Stars rating less than 0.302, both of which can be considered as very low scores.

| Quantile | HEI-2010 score | Guiding Stars rating |
| --- | --- | --- |
| 0% | 3.323 | 0 |
| 25% | 38.400 | 0.302 |
| 50% | 48.185 | 0.542 |
| 75% | 57.565 | 0.973 |
| 100% | 98.898 | 3 |
| Mean | 49.174 | 0.664 |
| Number of households | 2,218 | 2,218 |

*Discussion
282: Replace much with many*

**Response**: Thanks! We changed this and please see line 315.

*How will the proposed change affect the less nutrition oriented households? The discussion addresses the impact on nutrition-oriented households only. This needs to be discussed further in the discussion and conclusion.*

**Response**: Thanks for this question. For less nutrition-oriented households, SNAP restrictions might lead to improved nutritional outcomes and better health outcomes if they do not fully compensate by using cash income to purchase the SNAP-ineligible unhealthy foods. We added this in the “Discussion” and “Conclusions” section. Please see lines 327-323, and lines 388-392. *References:
Check that the article titles are in lower case.*

**Response**: Thanks for pointing this out. We revised the references to change the articles’ titles to lower case.
